# Health-related quality of life is an independent predictor of mortality and hospitalisations in transthyretin amyloid cardiomyopathy: a prospective cohort study

**DOI:** 10.1007/s11136-024-03723-y

**Published:** 2024-08-06

**Authors:** Michael Poledniczek, Christina Kronberger, Robin Willixhofer, Nikita Ermolaev, Bernhard Cherouny, Theresa-Marie Dachs, René Rettl, Christina Binder-Rodriguez, Luciana Camuz Ligios, Bernhard Gregshammer, Andreas Anselm Kammerlander, Johannes Kastner, Jutta Bergler-Klein, Franz Duca, Roza Badr Eslam

**Affiliations:** Department of Internal Medicine II, Division of Cardiology, Medical University of Vienna, Währinger Gürtel 18-20, Vienna, 1090 Austria

**Keywords:** Health-related quality of life, Quality of life, Transthyretin amyloid cardiomyopathy, Quality of care, Patient-reported outcomes

## Abstract

**Purpose:**

Transthyretin amyloid cardiomyopathy (ATTR-CM) is associated with severely impaired health-related quality of life (HRQL). HRQL is an independent predictor of outcome in heart failure (HF), but data on patients with ATTR-CM is scarce. This study therefore aims to evaluate the association of HRQL with outcome in ATTR-CM.

**Methods:**

Patients from our prospective ATTR-CM registry were assessed using the Kansas City cardiomyopathy questionnaire (KCCQ), the Minnesota living with HF questionnaire (MLHFQ), and the EuroQol five dimensions questionnaire (EQ-5D). Cox regression analysis was utilised to assess the impact of HRQL on all-cause mortality.

**Results:**

167 patients [80 years; interquartile range (IQR): 76–84; 80.8% male] were followed for a median of 27.6 (IQR: 9.7–41.8) months. The primary endpoint of all-cause mortality was met by 43 (25.7%) patients after a median period of 16.2 (IQR: 9.1–28.1) months. In a univariate Cox regression for mortality, a 10-point change in the KCCQ implied a hazard ratio (HR) of 0.815 [95%-confidence interval (CI): 0.725–0.916; *p* = 0.001], in the EQ-5D VAS of 0.764 (95%-CI: 0.656–0.889; *p* < 0.001), and 1.163 (95%-CI: 1.114–1.433; *p* < 0.001) in the MLHFQ. After adjustment for established biomarkers of HF, all-cause mortality was predicted independently by the EQ-5D VAS (HR: 0.8; 95%-CI: 0.649–0.986; *p* = 0.037; per 10 points) and the MLHFQ (HR: 1.228; 95%-CI: 1.035–1.458; *p* = 0.019; per 10 points).

**Conclusion:**

HRQL is a predictor of outcome in ATTR-CM. The EQ-5D VAS and the MLHFQ predict survival independent of biomarkers of HF.

**Supplementary Information:**

The online version contains supplementary material available at 10.1007/s11136-024-03723-y.

## Abstract (plain language summary)

Patients with transthyretin amyloid cardiomyopathy, a condition causing heart failure and mostly seen in the elderly, suffer from shortness of breath and reduced maximum physical performance. Disease assessment is currently based on blood analysis for markers of heart failure. However, standardised patient questionnaires also allow to estimate disease severity. In this study, we analyse different standardised patient questionnaires for their ability to predict adverse events including death and heart failure-related hospitalisations. The analysis demonstrates that an increase of ten points in the Kansas City Cardiomyopathy questionnaire, a tool specifically designed for patients with heart failure, implies a reduction of mortality risk of close to 20%. Interestingly, even the very simple visual analogue scale, a quality-of-life measurement tool which asks the patient to rate their health on a scale from zero (worst) to one hundred (best) has demonstrated remarkable predictive utility. An increase of ten points on this scale resulted in a reduction of risk for death from any cause of almost a quarter. This analysis suggests that standardised patient questionnaires for the assessment of quality of life may play an important role in the evaluation of patients with transthyretin amyloid cardiomyopathy and estimation of prognosis.

**Supplementary Information:**

The online version contains supplementary material available at 10.1007/s11136-024-03723-y.

## Background

Transthyretin amyloidosis is an infiltrative disorder predominantly affecting the myocardium and peripheral nerves [1]. As misfolded transthyretin fibrils accumulate in tissues, patients suffer from symptoms of heart failure (HF) and polyneuropathy. Additional disease manifestations may include carpal tunnel syndrome, spinal canal stenosis and autonomous dysfunction [2–4]. Disease may occur hereditarily due to pathogenic variants in the transthyretin gene (variant ATTR, ATTRv) or as an acquired disorder of the elderly (wild-type ATTR, ATTRwt).

Health-related quality of life (HRQL) has been demonstrated to be significantly impaired in transthyretin amyloid cardiomyopathy (ATTR-CM) [2, 5, 6]. Determinants as well as prognostic implications of HRQL have previously been explored in the general HF population [7–11]. In these patients, HRQL is an independent predictor of mortality and adverse cardiac events [7]. While with regard to patients with ATTR-CM, HRQL has been described in detail, prognostic implications of HRQL have not yet been explored in the literature [2, 5, 6].

We provide a thorough insight into HRQL by analysing results from the EuroQol five dimensions questionnaire (EQ-5D), the Minnesota living with HF questionnaire (MLHFQ) and the Kansas City cardiomyopathy questionnaire (KCCQ) in ATTR-CM patients. All three tools have been thoroughly validated in various aetiologies of HF and are standard tools which are broadly applied in large-scale clinical trials [8, 12–15].

The aim of our study was to evaluate the association between HRQL and outcomes, i.e., all-cause mortality and a composite endpoint of all-cause mortality and HF-related hospitalisation. A secondary aim was to evaluate the difference in clinical parameters, HRQL, and outcomes in patients with ATTRwt and ATTRv and potential differences in HRQL between these two cohorts.

## Methods

### Setting

The present investigation was conducted within the scope of a prospective cardiac amyloidosis registry at the Medical University of Vienna, Department of Internal Medicine II – Division of Cardiology. The registry was approved by the local ethics committee (#796/2010) and was implemented in accordance with the declaration of Helsinki. All patients provided informed consent per personal signature prior to inclusion into the registry. All data is acquired by study personnel and, following pseudonymisation, stored locally in a dedicated electronic database.

### Subjects and study design

All patients were recruited between April 2018 and April 2023 from the clinic’s dedicated cardiac amyloidosis out-patient clinic. Patients were screened for eligibility and included if the predefined inclusion criteria were met: (I) a definite diagnosis of ATTR-CM, (II) consent to perform repeated assessments as described below, (III) ability to read, understand, and comply with the study requirements, (IV) evidence of at least one completed measurement of HRQL, and (V) a personally signed informed consent form.

A diagnosis of ATTR-CM was established either by means of endomyocardial biopsy, subsequent congo-red staining and immuno-histochemistry, or utilising the non-invasive diagnostic algorithm by Gillmore et al. [16] In patients with moderate or strong cardiac ^99m^Technetium-based tracer uptake in bone scintigraphy (Perugini grade [17] ≥ 2) and exclusion of light chain amyloidosis by means of serum and urine electrophoresis and immunofixation, a diagnosis of ATTR-CM was made. For the discrimination of the wild-type ATTR (ATTRwt) amyloidosis and variant ATTR (ATTRv) amyloidosis, genetic sequencing was offered to and accepted by all patients who had received a diagnosis of ATTR-CM.

Disease monitoring was performed in accordance with expert consensus recommendations published by Garcia-Pavia et al. [18] and the Austrian social security guidelines for the approval of the ATTR-CM therapeutic tafamidis.

### Evaluation of health-related quality of life

HRQL was evaluated using two HF-specific questionnaires and one non-specific questionnaire. During routine in-clinic visits, patients completed the German versions of the respective questionnaires self-sufficiently.

### Kansas city cardiomyopathy questionnaire

The KCCQ is a HF-specific HRQL measurement tool [12]. It is widely used in the context of clinical trials and its validity and reliability have been extensively studied. [19, 20]. Importantly, the KCCQs responsiveness to changes in disease status have been explored in various entities of HF irrespective of ejection fraction and has been demonstrated to predict all-cause mortality and HF-related hospitalisations [19–22]. To reduce time to completion and increase patients’ acceptance of HRQL questionnaires, a shortened 12-item version of the KCCQ has been developed, which was used in this investigation [23]. This abbreviated version of the KCCQ has been demonstrated to retain the original 23-item questionnaire’s psychometric properties, increase response rates and reduce time exposure [23]. After completion, four sub-scores and one summary score are calculated, which was used throughout this analysis [12].

### Minnesota living with heart failure questionnaire

The MLHFQ was also designed specifically for patients with HF. It has been extensively studied and is widely used in both research and clinical practice [13, 24–26]. The MLHFQ takes about two to three minutes to complete and the score provided is responsive to changes in patients’ disease status [13, 24, 25, 26]. Patients are asked to rate their disease-related impairment to their every-day life on a scale from 0 (no impairment) to 5 (most severe impairment) in the context of 21 situations ranging from leisure activities to anxiety and depression, financial stability and sexual well-being [13, 24, 27]. The MLHFQ score is scaled from 0 to 105, where higher values indicate worse HRQL [13]. In a direct comparison, Yee et al. have found a non-significant advantage for the KCCQ with regard to predictive utility [28]. However, this has been demonstrated primarily for patients with HF with reduced ejection fraction and the specific advantages of either tool remain a matter of discussion [28].

### EuroQol five dimensions questionnaire

The EQ-5D is a non-disease specific measurement tool of general health status and HRQL. The EQ-5D assesses impairment in five dimensions (mobility, self-sufficiency, activities of every-day life, pain, anxiety & depression) in three levels (EQ-5D-3L) ranging from no, moderate to severe impairment [29]. In the EQ-5D, a score of 1 indicates no impairments to HRQL [29]. For each denoted level of impairment, a number taken from geographically specific validation sets is subtracted [29]. For this analysis, the validation set for Europe was utilised [30]. Owing to its simplicity, the EQ-5D can usually be completed in less than one minute. In addition, patients are asked to rate their overall health status on a visual analogue scale (VAS) ranging from 0 (worst imaginable) to 100 (best imaginable).

### Laboratory evaluations and biomarkers of heart failure

As part of in-clinic visits, a complete hemogram, blood chemistry, and biomarkers of HF [N-terminal prohormone of brain natriuretic peptide (NT-proBNP), troponin t (TnT), and creatinine / estimated glomerular filtration rate (eGFR)] were assessed in the clinic’s central laboratory institute (Department of Laboratory Medicine, Medical University of Vienna, Vienna). The eGFR was calculated using the chronic kidney disease epidemiology collaboration formula [31].

### Medical history

Medical history of participants was assessed by a trained physician at baseline. In addition, electronic health records were analysed, if patients failed to recall previous procedures or diagnosis. Significant coronary artery disease was assumed in patients with a history of percutaneous coronary intervention or coronary artery bypass grafting. Arterial hypertension was defined as history of blood pressure in excess of 140 mmHg systole or 90 mmHg diastole [32]. Diabetes mellitus was defined as a level of glycated haemoglobin ≥ 6.5% without anti-diabetic medication [33]. A history of carpal tunnel syndrome was assumed in patients who had either electromyographic evidence of carpal tunnel syndrome or who had undergone either uni- or bilateral carpal tunnel decompression surgery.

### Study endpoints

A primary endpoint of all-cause mortality was used for the present investigation. Mortality data were acquired using periodic queries on the national statistics authority (Statistik Austria), telephone interviews, online research and in- and out-of-centre medical documentation. In analogy to a study by Ravera et al. [19], a secondary endpoint of all-cause mortality and HF-related hospitalisations was explored using electronic health records and patient interviews. Hospitalisations were assumed to be HF-related when (1) the admission was due to dyspnoea, peripheral or central oedema, (2) symptoms were resolved by HF-specific therapy and/or forced diuresis, and (3) in absence of a diagnosis other than HF which the patient’s symptoms are attributed to.

### Statistics

Categorial variables are presented as numbers and percentages while continuous variables are presented as mean and standard deviation (SD) or median and interquartile range (IQR) depending on normal distribution, which was assessed using the Kolmogorov-Smirnov test. Baseline characteristics were compared between the ATTRwt and the ATTRv sub cohort using the chi-square test and the t-test or the Mann-Whitney-U test as applicable. For the correlation between HRQL scores and biomarkers of HF, the Pearson correlation coefficient (PC) was calculated.

To test for prognostic implications of the HRQL scores, univariate Cox proportional hazard regression analysis was performed. In a multivariable Cox proportional hazard regression model, HRQL scores were adjusted for levels of NT-proBNP, troponin t, and the estimated glomerular filtration rate, as these variables constitute the most widely used disease specific staging systems in ATTR-CM [34, 35].

Both the univariate as well as the multivariable model were applied for the primary endpoint of all-cause mortality as well as a composite endpoint of all-cause mortality and HF-hospitalisations. In addition to the hazard ratios (HR) per unit of the respective questionnaire, we also report 10-point increments for the KCCQ, the MLHFQ, and the EQ-5D VAS as previous studies have identified a range of 5–10 points as the minimal clinically important difference in individuals with HF for outcome prognosis [22, 36].

In case of missing questionnaire scores, the respective patients were excluded from the analysis for the specific questionnaire. Statistical significance was assumed with a confidence interval of 95% and a P-value of < 0.05, respectively. All statistical analysis was performed using IBM SPSS Statistics version 29 (IBM Corporation, Armonk, NY, USA) [37].

## Results

### Baseline characteristics

**Fig. 1:**
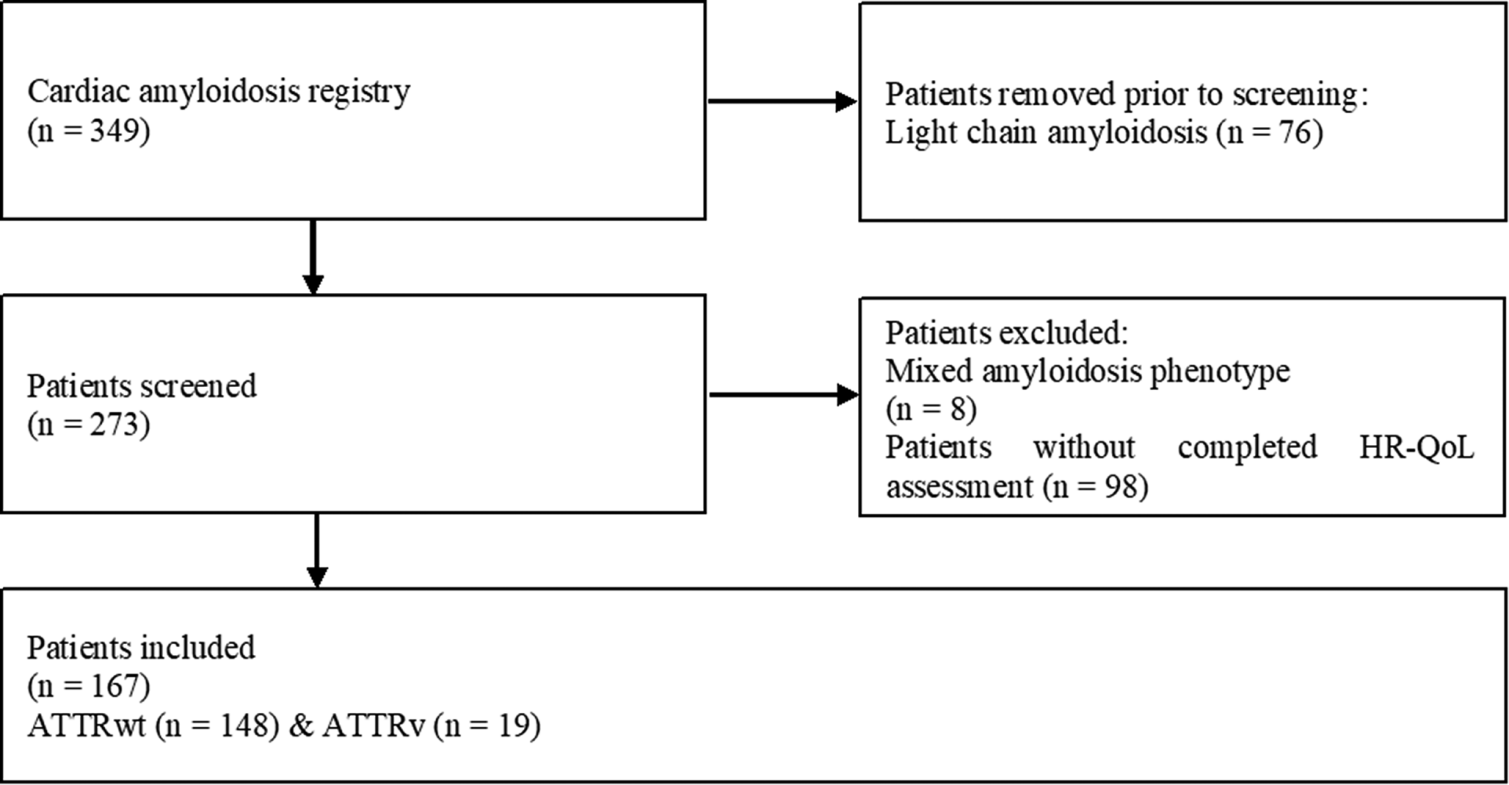
The patient recruitment process. *ATTRv indicates variant transthyretin amyloid; ATTRwt, wild-type transthyretin amyloid; HR-QoL, health-related quality of life*

The entire cohort comprised 167 patients, 19 (11.4%) of which had been diagnosed with a pathogenic variant in the transthyretin gene. Patients mean age was 79.0 (SD: 7.6) years at inclusion with significant differences between the ATTRwt amyloidosis (80.6; SD: 5.8) and the ATTRv amyloidosis (66.5; SD: 8.3; *p* < 0.001) cohorts. Patients were followed for median period of 27.6 (IQR: 9.7–41.8) months. The detailed recruitment process is depicted in Fig. 1.

Clinically symptomatic disease defined as a New York Heart Association (NYHA) stage ≥ 2 was present in 119 (74.4%) of patients without significant differences between cohorts. Biomarkers of HF were elevated with a median NT-proBNP of 2579 pg/mL (IQR: 1361–4438) and a median TnT of 51 ng/L (IQR: 35–74). The median eGFR was 53.7 ml/min/1.73m^2^ [IQR: 41.0–67.2; 52.5 (IQR: 39.5–65.2) vs. 60.7 (IQR: 56.7–73.6); *p* = 0.003]. Statistically significant differences between the ATTRwt and the ATTRv cohort were observed in the mean systolic blood pressure, and the rate of history of atrial fibrillation and carpal tunnel syndrome. The detailed baseline characteristics of the patient cohort are depicted in Table 1.

**Table 1:** The patient cohorts’ baseline characteristics

Variable	Entire cohort (*n* = 167)	ATTRwt (*n* = 148)	ATTRv (*n* = 19)	*P-value*
Demographics				
Age, years, mean (SD)	79.0 (7.6)	80.6 (5.8)	66.5 (8.3)	***< 0.001***
Male sex, n (%)	135 (80.8%)	121 (81.8%)	14 (73.7%)	*0.40*
**Outcome**				
All-cause mortality, n (%)	43 (25.7%)	39 (26.4%)	4 (21.1%)	*0.62*
*Days to event, mean (SD)*	539 (348)	553 (353)	401 (298)	*0.40*
Combined endpoint, n (%)	62 (37.1%)	55 (37.2%)	7 (36.8%)	*0.98*
*Days to event, median (IQR)*	405 (159–719)	416 (155–719)	197 (186–806)	*0.66*
**Clinical parameters**				
BMI, median (IQR)	25.2 (23.0–27.5)	25.1 (22.9–27.3)	25.4 (23.9–29.1)	*0.12*
Heart rate, bpm, median (IQR)	70 (62–79)	70 (62–79)	72 (64–80)	*0.63*
BP systolic, mmHg, mean (SD)	134 (19)	136 (19)	122 (15)	***0.005***
BP diastolic, mmHg, median (IQR)	78 (70–85)	79 (70–85)	75 (69–80)	*0.31*
NT-proBNP, pg/mL, median (IQR)	2579 (1361–4438)	2569 (1414–4438)	2633 (720–4308)	*0.76*
TnT, ng/L, median (IQR)	51 (35–74)	51 (35–75)	50 (32–58)	*0.48*
Creatinine, mg/dL, median (IQR)	1.24 (1.01–1.50)	1.25 (1.01–1.56)	1.22 (1.05–1.31)	*0.21*
eGFR, mL/min/1.73m^2^, mean (SD)	54.1 (19.4)	52.7 (19.5)	65.3 (14.3)	***0.003***
C-reactive protein, mg/dL, median (IQR)	0.19 (0.08–0.42)	0.18 (0.08–0.36)	0.30 (0.11–0.55)	*0.26*
**Comorbidities and medical history**				
Atrial fibrillation, n (%)	101 (61.2%)	97 (66.0%)	4 (22.2%)	***< 0.001***
Significant coronary artery disease, n (%)	41 (25.2%)	37 (25.5%)	4 (22.2%)	*0.76*
Arterial hypertension, n (%)	117 (71.0%)	107 (72.8%)	10 (55.6%)	*0.13*
Diabetes mellitus, n (%)	36 (22.2%)	33 (22.9%)	3 (16.7%)	*0.55*
Carpal tunnel syndrome, n (%)	80 (48.8%)	65 (44.8%)	15 (78.9%)	***0.005***
Intervention for valvular heart disease, n (%) *Aortic valve* *Mitral valve* *Tricuspid valve*	22 (13.4%) 17 (10.4%) 7 (4.3%) 4 (2.4%)	22 (15.0%) 17 (11.6) 7 (4.8%) 4 (2.7%)	0 (0.0%) 0 (0.0%) 0 (0.0%) 0 (0.0%)	*0.13*
**Transthyretin amyloid cardiomyopathy staging**				
NYHA, n (%) *1* *2* *3* *4*	41 (25.6%) 69 (43.1%) 50 (31.3%) 0 (0.0%)	37 (26.2%) 60 (42.6%) 44 (31.2%) 0 (0.0%)	4 (21.1%) 9 (47.4%) 6 (31.6%) 0 (0.0%)	*0.88*
MAYO, n (%) *1* *2* *3*		40 (36.7%) 31 (28.4%) 38 (34.9%)		
NAC, n (%) *1* *2* *3*	69 (46.9%) 44 (29.9%) 34 (23.1%)	59 (45.0%) 38 (29.0%) 34 (26.0%)	10 (65.5%) 6 (37.5%) 0 (0.0%)	*0.07*
COLUMBIA, n (%) *1* *2* *3*	47 (32.9%) 61 (42.7%) 35 (24.5%)	40 (32.0%) 54 (43.2%) 31 (24.8%)	7 (38.9%) 7 (38.9%) 4 (22.2%)	*0.84*
**Transthyretin amyloid cardiomyopathy and heart failure medication**				
Tafamidis, n (%) *20 mg* *61 mg*	75 (46.3%) 7 (4.3%) 68 (42.0%)	70 (49.0%) 7 (4.9%) 63 (44.1%)	5 (26.3%) 0 (0.0%) 5 (26.3%)	*0.06* *0.32* *0.14*
Patisiran, n (%)	2 (1.2%)	0 (0.0%)	2 (10.5%)	***< 0.001***
Inotersen, n (%)	2 (1.2%)	0 (0.0%)	2 (10.5%)	***< 0.001***
Beta receptor antagonists, n (%)	76 (46.9%)	69 (48.3%)	7 (36.8%)	*0.35*
ACEi / AT1i / ARNi, n (%)	81 (50.0%)	72 (50.3%)	9 (47.4%)	*0.81*
MRA, n (%)	71 (43.8%)	63 (44.1%)	8 (42.1%)	*0.87*
SGLT-2i, n (%)	9 (5.6%)	8 (5.6%)	1 (5.3%)	*0.95*
**Health-related quality of life**				
EQ-5D score, mean (SD; n; available %)	0.690 (0.220; 163; 97.6%)	0.694 (0.221; 144; 97.3%)	0.661 (0.219; 19; 100%)	*0.50*
EQ-5D VAS, mean (SD; n; available %)	62 (22; 156; 93.4%)	61 (22; 138; 93.2%)	63 (21; 18; 94.7%)	*0.74*
KCCQ, mean (SD; n; available %)	61.3 (25.5; 167; 100%)	61.2 (25.6; 148; 100%)	61.7 (25.6; 19; 100%)	*0.98*
MLHFQ, mean (SD; n; available %)	32.0 (23.6; 157; 94.0%)	31.6 (23.6; 138; 93.2%)	35.2 (24.0; 19; 100%)	*0.61*

*ACEi indicates angiotensin converting enzyme inhibitor; ARNi, angiotensin receptor neprilysin inhibitor; AT1i, angiotensin receptor 1 inhibitor; ATTRv, variant transthyretin amyloid; ATTRwt, wild-type transthyretin amyloid; BMI, body-mass index; BP, blood pressure; bpm, beats per minute; COLUMBIA, Columbia university staging system* [34]; *eGFR, estimated glomerular filtration rate; EQ-5D, EuroQol five dimensions questionnaire; HF heart failure; IQR, interquartile range; KCCQ, Kansas City cardiomyopathy questionnaire; MAYO, Mayo Clinic staging system* [35]; *MLHFQ, Minnesota living with heart failure questionnaire; mmHg, millimetres of mercury; MRA, mineralocorticoid receptor antagonist; NAC, United Kingdom National Amyloidosis Centre staging system* [36]; *NT-proBNP, N-terminal prohormone of brain natriuretic peptide; NYHA, New York Heart Association stage; SD, standard deviation; SGLT-2i, sodium-glucose cotransporter 2 inhibitor; TnT, Troponin T; VAS, visual analogue scale*

For normally distributed categorial variables, mean and standard deviation are reported and the t-test was used for comparison; for non-normally distributed variables, median and standard deviation are reported and the Mann-Whitney-U test was utilised

HRQL scores are conventionally reported as mean (SD), however, due to non-normal distribution, the Mann-Whitney-U test was utilised

In comparison to the 98 patients who have been screened for eligibility but have not been included in the final analysis due to missing HRQL measurements, no statistically significant differences with regard to age, sex, biomarkers of HF and kidney function, and distribution of NAC stages were observed.

**Fig. 2:**
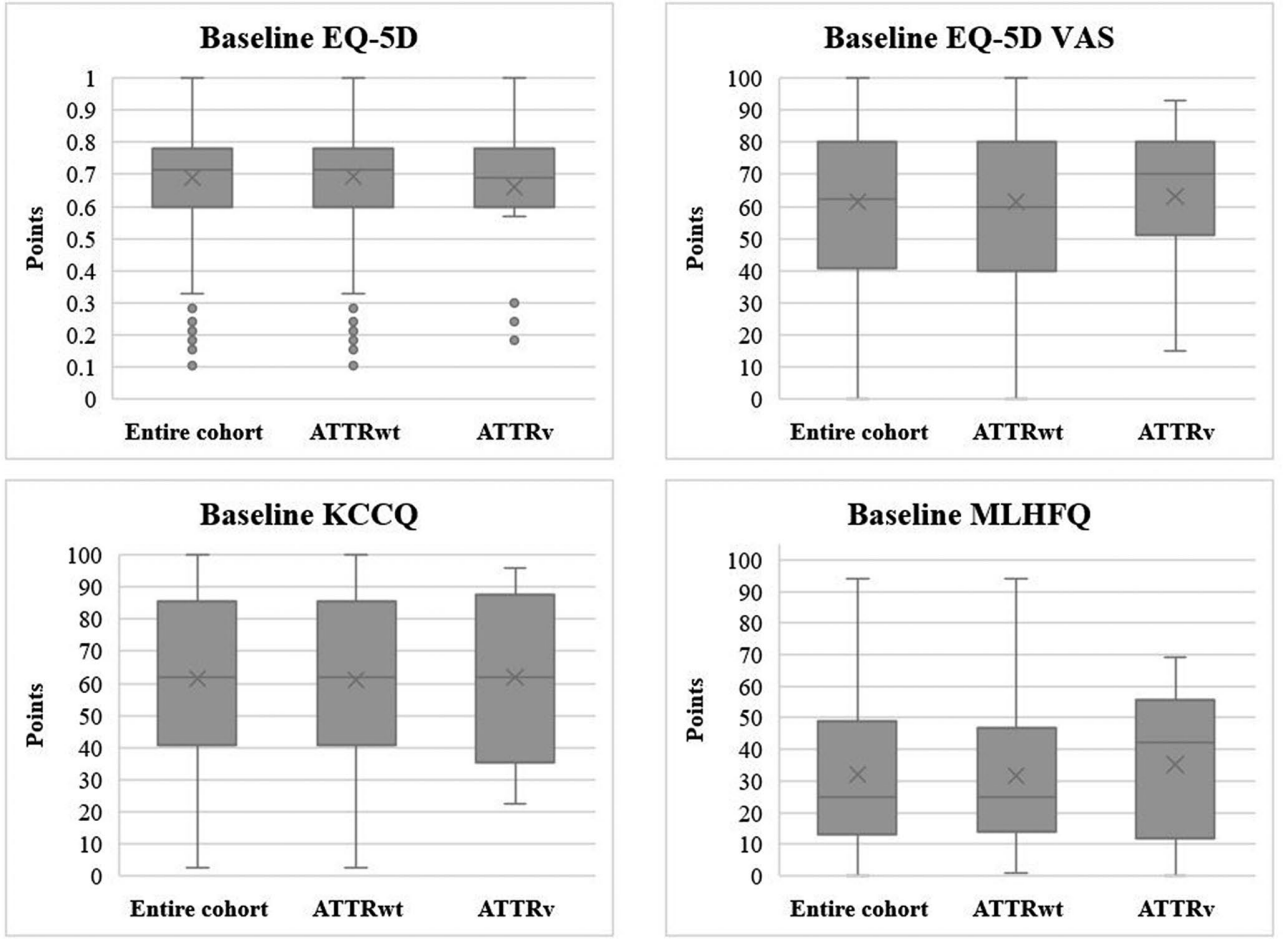
Health-related quality of life scores at baseline. *ATTRv indicates variant transthyretin amyloid; ATTRwt, wild-type transthyretin amyloid; EQ-5D, EuroQol five dimensions questionnaire; KCCQ, Kansas City cardiomyopathy questionnaire; MLHFQ, Minnesota living with heart failure questionnaire; VAS, visual analogue scale*

The mean HRQL scores were 0.690 (SD: 0.220) for the EQ-5D, 62 (SD: 22) for the EQ-5D VAS, 61.3 (SD: 25.5) for the KCCQ, and 32.0 (SD: 23.9) for the MLHFQ. No significant differences were observed between the cohorts. All results can be found in Table 1 and Fig. 2.

### Correlation analysis

In the Pearson correlation analysis between all HRQL scores, the correlation between the KCCQ and the MLHFQ was highest [PC: -0.887; *p* < 0.001]. The lowest level of correlation between any HRQL scores was observed between the EQ-5D score and the EQ-5D VAS (PC: 0.531; *p* < 0.001). Levels of correlation between biomarkers of HF and HRQL scores was generally low to moderate. All results are listed in Table 2.

**Table 2:** Correlation analysis of health-related quality of life scores, biomarkers of heart failure, and loop diuretic prescription

Variable	EQ-5D score	EQ-5D-VAS	KCCQ	MLHFQ	NT-proBNP	TnT
EQ-5D-VAS *P-value*	0.531 ***< 0.001***					
KCCQ *P-value*	0.631 ***< 0.001***	0.641 ***< 0.001***				
MLHFQ *P-value*	-0.714 ***< 0.001***	-0.622 ***< 0.001***	-0.887 ***< 0.001***			
NT-proBNP *P-value*	-0.046 *0.58*	-0.206 ***0.016***	-0.219 ***0.007***	0.159 *0.06*		
TnT *P-value*	-0.075 *0.42*	-0.188 ***0.042***	-0.212 ***0.018***	0.179 *0.06*	0.742 ***< 0.001***	
eGFR *P-value*	0.073 *0.38*	0.181 ***0.030***	0.340 ***< 0.001***	-0.180 ***0.031***	-0.501 ***< 0.001***	-0.523 ***< 0.001***

*eGFR indicates estimated glomerular filtration rate; EQ-5D, EuroQol five dimensions questionnaire; KCCQ, Kansas City cardiomyopathy questionnaire; MLHFQ, Minnesota living with heart failure questionnaire; NT-proBNP, N-terminal prohormone of brain natriuretic peptide; VAS, visual analogue scale; TnT, Troponin T*

### Outcome – all-cause mortality

The primary endpoint of all-cause mortality occurred in 43 (25.7%) patients after a median period of 16.2 (IQR: 9.1–28.1) months. After 1 and 2 years, respectively, 118 (88.1%) and 90 (75.6%) of patients were alive.

In the univariate Cox regression, NT-proBNP [HR: 1.009 per 100 pg/mL; 95%-confidence interval (CI): 1.006–1.013; *p* < 0.001], TnT (HR: 1.009; 95%-CI: 1.005–1.013; *p* < 0.001), and eGFR (HR: 0.962; 95%-CI: 0.946–0.979; *p* < 0.001) proved significant predictors of all-cause mortality. The tested scores of HRQL also exhibited significant predictive utility with the exception of the EQ-5D score. The EQ-5D VAS implied a HR of 0.764 (95%-CI: 0.656–0.889; *p* < 0.001), the KCCQ of 0.815 (95%-CI: 0.725–0.916; *p* = 0.001), and the MLHFQ of 1.163 (95%-CI: 1.114–1.433; *p* < 0.001) per 10 points, respectively. All results are depicted in the supplementary table S1 and figure S1.

In a multivariable model, the EQ-5D VAS, the KCCQ, and the MLHFQ were adjusted for NT-proBNP, TnT, and eGFR. After adjustment, the EQ-5D VAS implied a HR of 0.8 (95%-CI: 0.649–0.986; *p* = 0.037) and the MLHFQ of 1.228 (95%-CI: 1.035–1.458; *p* = 0.019). The KCCQ failed to reach the predefined level of statistical significance (HR: 0.886; 95%-CI: 0.760–1.032; *p* = 0.12). The detailed results are shown in Table 3 and supplementary figure S1. When adjusted for clinical characteristics including age, sex, ATTRwt and treatment status, and NT-proBNP, all HRQL scores also provided comparable independent predictive value for all-cause mortality as depicted in the supplementary table S2.

**Table 3:** Cox regression for all-cause mortality after adjustment for biomarkers of heart failure

Variable	Hazard ratio	95%-CI	*P*-value
EQ-5D-VAS *10 points*	0.978 *0.800*	0.958–0.999 *0.649–0.986*	***0.037***
KCCQ *10 points*	0.988 *0.886*	0.973–1.003 *0.760–1.032*	*0.12*
MLHFQ *10 points*	1.021 *1.228*	1.003–1.038 *1.035–1.458*	***0.019***

*CI indicates confidence interval; EQ-5D, EuroQol five dimensions questionnaire; KCCQ, Kansas City cardiomyopathy questionnaire; MLHFQ, Minnesota living with heart failure questionnaire; VAS, visual analogue scale*

### Outcome - all-cause mortality and heart failure-related hospitalisation

For the combined endpoint of all-cause mortality and HF-related hospitalisation, a total of 62 events were recorded and 37.1% of patients met this secondary endpoint after a median period of 13.5 (IQR: 5.3–24.5) months.

In a univariate Cox regression analysis, biomarkers of HF (TnT: 1.007; 95%-CI: 1.003–1.010; *p* < 0.001; NT-proBNP: 1.009; 95%-CI: 1.006–1.012; *p* < 0.001) as well as eGFR (HR: 0.963; 95%-CI: 0.949–0.977; *p* < 0.001) provided predictive utility. All HRQL scores predicted the combined endpoint with a HR of 0.334 (95%-CI: 0.117–0.954; *p* = 0.041) for the EQ-5D score, 0.755 (95%-CI: 0.666–0.857; *p* < 0.001) on the EQ-5D VAS, 0.794 (95%-CI: 0.721–0.874; *p* < 0.001) on the KCCQ, and 1.278 (95%-CI: 1.149–1.422; *p* < 0.001) for the MLHFQ per 10 points, respectively. All results are depicted in the supplementary table S3 and figure S2.

In the multivariable Cox regression model after adjustment for NT-proBNP, TnT, and eGFR, all scores reached statistical significance (EQ-5D score HR: 0.262; 95%-CI: 0.076–0.910; *p* = 0.035; EQ-5D VAS HR: 0.778; 95%-CI: 0.662–0.915; *p* = 0.002; KCCQ HR: 0.862; 95%-CI: 0.766–0.970; *p* = 0.014; MLHFQ 1.256; 95%-CI: 1.092–1.444; *p* = 0.001). Detailed results are shown in Table 4 and the supplementary figure S2. In multivariable Cox regression adjusted for clinical characteristics and NT-proBNP (table S4), similar independent predictive value for the combined endpoint is also demonstrated for all tested HRQL scores.

**Table 4:** Cox regression for the combined endpoint of all-cause mortality and heart failure-related hospitalisations after adjustment for biomarkers of heart failure

Variable	Hazard ratio	95%-CI	*P-value*
EQ-5D score	0.262	0.076–0.910	***0.035***
EQ-5D-VAS *10 points*	0.975 *0.778*	0.960–0.991 *0.662–0.915*	***0.002***
KCCQ *10 points*	0.985 *0.862*	0.974–0.997 *0.766–0.970*	***0.014***
MLHFQ *10 points*	1.023 *1.256*	1.009–1.037 *1.092–1.444*	***0.001***

*CI indicates confidence interval; EQ-5D, EuroQol five dimensions questionnaire; KCCQ, Kansas City cardiomyopathy questionnaire; MLHFQ, Minnesota living with heart failure questionnaire; VAS, visual analogue scale*

## Discussion

The results presented demonstrate that HRQL assessed using the EQ-5D VAS, the KCCQ, and the MLHFQ is a predictor of outcome in ATTR-CM. Our multivariable regression models indicate that this predictive value is independent of established biomarkers of HF in ATTR-CM.

While our analysis was not designed to differentiate between distinct questionnaire scores, the EQ-5D score was the only score which failed to imply predictive value with regard to all-cause mortality. Interestingly, the EQ-5D VAS, an extremely simplistic HRQL measurement tool, demonstrated HRs numerically quite similar to that of more complex questionnaires.

Although established in the general HF population, to the best of our knowledge, prognostic implications of HRQL have not yet been explored in the context of ATTR-CM specifically. Importantly, our real-world findings have implications both for disease staging and monitoring and may also aid the design of clinical trials in the future. As the tested HRQL questionnaires are applicable without the need for neither a physician nor extensive laboratory equipment required for the assessment of biomarkers of HF, these tools may prove valuable in the context of telehealth applications, remote disease monitoring, and early detection of disease progression and decompensation.

Compared to previously published ATTR-CM cohorts, our patients were slightly older, but demonstrated similar HRQL [5, 6, 38]. Patients with ATTRwt tended to be older and presented more frequently with history of atrial fibrillation and worse kidney function. However, in contrast to an earlier study [6], we have not observed relevant differences between our ATTRwt and our ATTRv patients with regard to HRQL. This may be attributed to a relatively small ATTRv cohort size. While positive effects of tafamidis on HRQL have been previously described in clinical studies [38], we have demonstrated the prognostic implications of HRQL in a non-selected real-world sample of patients with ATTR-CM independent of tafamidis treatment status at baseline (data not shown).

Patients with ATTR-CM appear to report more severely impaired HRQL than patients with heart failure with both preserved [8] and reduced ejection fraction [39], and hypertrophic cardiomyopathy [6, 40]. While the reasons have not yet been explored in detail, it may be hypothesised that the multi-system involvement often observed in ATTR-CM could be a significant factor [6]. Future studies will need to describe the yet unexplored influence of both polyneuropathy and autonomic dysfunction on HRQL in ATTR-CM in order to gain a better understanding of patients’ limitations and offer treatment where available.

Interestingly, while the tested scores with the exception of the EQ-5D score correlated quite well among each other, the level of correlation between biomarkers of HF, the eGFR, and HRQL outcome measurement tools’ scores was low. We suspect that this may be due to socio-psychological factors, disease awareness, and coping strategies which may limit subjective disease severity in some patients despite advanced disease. Future studies will therefore need to evaluate which strategies and socio-psychological factors and interventions improve HRQL in patients with ATTR-CM.

In rare diseases, and especially in the case of ATTR-CM, the selection and design of HRQL measurement tools may be complicated by disease-specific symptoms [41]. In practice, specialised centres may choose to apply more than one tool to assess HRQL comprehensively. While we have not assessed tools on anxiety or depression specifically, in clinical practice, such tools may also have additional value for comprehensive patient care. In healthcare systems strained for resources, the standardised assessment of HRQL using questionnaires may also have the potential to improve patient care and the investigation of symptoms. This is especially relevant with regard to disease manifestations concerning sexuality, intimacy, and concomitant psychologic ailments including depression, where some patients may refrain from reporting impairment in a direct conversation with a physician.

### Limitations and potential bias

A number of limitations of this study need to be considered. First, as with every observational study, unidentified residual confounding factors cannot be fully excluded despite scientific rigour. As reasons for failing or declining to complete HRQL questionnaires have not been analysed in our study, eventual bias with regard to patient selection cannot be fully excluded. Due to the prospective nature of our registry, an objective primary endpoint of all-cause mortality, and event adjudication blinded to the parameters of interest, we have sought to minimise the introduction of potential bias during data analysis. However, ATTR-CM remains a disease characterised by varying presentation and severity, and the relatively small and selected patient cohort presenting to our tertiary referral centre may be not fully representative of the entire ATTR-CM population. Therefore, the findings presented ought to be confirmed in a larger, and ideally multi-centre, cohort of patients with ATTR-CM.

## Conclusion

HRQL is a predictor of mortality and HF-related hospitalisations in ATTR-CM. The EQ-5D and the MLHFQ predict all-cause mortality independent of biomarkers of HF and all scores tested demonstrated independent predictive value for the combined endpoint of all-cause mortality and HF-related hospitalisations.

## Electronic supplementary material

Below is the link to the electronic supplementary material.

Supplementary Material 1

## Data Availability

The data underlying this article will be shared on reasonable request to the corresponding author.

## Abbreviations

ACEi: Angiotensin converting enzyme inhibitor
ARNi: Angiotensin receptor neprilysin inhibitor
AT1i: Angiotensin receptor 1 inhibitor
ATTR-CM: Transthyretin amyloid cardiomyopathy
ATTRv: Variant transthyretin amyloid
ATTRwt: Wild-type transthyretin amyloid
BMI: Body-mass index
BP: Blood pressure
COLUMBIA: Columbia university staging system
eGFR: Estimated glomerular filtration rate
EQ-5D: EuroQol five dimensions questionnaire
HF: Heart failure
HR: Hazard ratio
HRQL: Health-related quality of life
IQR: Interquartile range
KCCQ: Kansas City cardiomyopathy questionnaire
MAYO: Mayo Clinic staging system
MLHFQ: Minnesota living with heart failure questionnaire
MRA: Mineralocorticoid receptor antagonist
NAC: United Kingdom National Amyloidosis Centre staging system
NT-proBNP: N-temrinal prohormone of brain natriuretic peptide
NYHA: New York Heart Association staging system
PC: Pearson correlation coefficient
SD: Standard deviation
SGLT-2i: Sodium-glucose cotransporter 2 inhibitor
TnT: Troponin t
VAS: Visual analogue scale
