# Supplementary material for: Health-related quality of life is an independent predictor of mortality and hospitalisations in transthyretin amyloid cardiomyopathy: a prospective cohort study
Source: Qual Life Res. 2024 Aug 6;33(10):2743–53. doi: 10.1007/s11136-024-03723-y (PMC11452430; doi:10.1007/s11136-024-03723-y)
Supplement: Supplementary file 1 — Supplementary Material 1 [file 11136_2024_3723_MOESM1_ESM.docx]

STROBE Statement—checklist of items that should be included in reports of observational studies

|  | Item No. | Recommendation | Page  No. | Relevant text from manuscript |
| --- | --- | --- | --- | --- |
| **Title and abstract** | 1 | (*a*) Indicate the study’s design with a commonly used term in the title or the abstract | 3 | *Patients from our prospective ATTR-CM registry…* |
|  |  | (*b*) Provide in the abstract an informative and balanced summary of what was done and what was found | 3 | *Purpose. Transthyretin amyloid cardiomyopathy (ATTR-CM) is associated with severely impaired health-related quality of life (HR-QoL). HR-QoL is an independent predictor of outcome in heart failure (HF), but data on patients with ATTR-CM is scarce. This study therefore aims to evaluate the association of HR-QoL with outcome in ATTR-CM.*  *Methods. Patients from our prospective ATTR-CM registry were assessed using the Kansas City cardiomyopathy questionnaire (KCCQ), the Minnesota living with HF questionnaire (MLHFQ), and the EuroQol five dimensions questionnaire (EQ-5D).*  *Results. 167 patients (79 ± 8 years; 80.8% male; 11.4% variant ATTR-CM) were included and followed for a median of 27.6 [interquartile range (IQR): 9.7 – 41.8] months. The primary endpoint of all-cause mortality was met by 43 (25.7%) patients after a median period of 16.2 (IQR: 9.1 – 28.1) months.*  *In a univariate Cox regression for all-cause mortality, a 10-point change in the KCCQ implied a hazard ratio (HR) of 0.815 [95%-confidence interval (CI): 0.725 – 0.916; p = 0.001], in the EQ-5D VAS of 0.764 (95%-CI: 0.656 – 0.889; p = <0.001), and 1.163 (95%-CI: 1.114 – 1.433; p = <0.001) in the MLHFQ. After adjustment for established biomarkers of HF, all-cause mortality was predicted independently by the EQ-5D VAS (HR: 0.8; 95%-CI: 0.649 – 0.986; p = 0.037; per 10 points) and the MLHFQ (HR: 1.228; 95%-CI: 1.035 – 1.458; p = 0.019; per 10 points).*  *Conclusion. HR-QoL is valid predictor of outcome in ATTR-CM. The EQ-5D VAS and the MLHFQ predict survival independent of biomarkers of HF.* |
| Introduction | | | |  |
| Background/rationale | 2 | Explain the scientific background and rationale for the investigation being reported | 7 | *Both in patients with HF with reduced ejection fraction as well as in those with preserved ejection fraction, HR-QoL is an independent predictor of mortality and adverse cardiac events. With regard to patients with ATTR-CM, however, data on prognostic implications of HR-QoL is lacking.* |
| Objectives | 3 | State specific objectives, including any prespecified hypotheses | 7 | *We therefore sought to provide a thorough insight into HR-QoL in ATTR-CM by analysing the prognostic utility of the EuroQol five dimensions questionnaire (EQ-5D), the Minnesota living with HF questionnaire (MLHFQ) and the Kansas City cardiomyopathy questionnaire (KCCQ) in patients with ATTR-CM.* |
| Methods | | | |  |
| Study design | 4 | Present key elements of study design early in the paper | 7 – 12 | *Please refer to the Methods section* |
| Setting | 5 | Describe the setting, locations, and relevant dates, including periods of recruitment, exposure, follow-up, and data collection | 7 - 8 | *All patients were recruited between April 2018 and April 2023 from the clinic’s dedicated cardiac amyloidosis out-patient clinic.* |
| Participants | 6 | (*a*) *Cohort study*—Give the eligibility criteria, and the sources and methods of selection of participants. Describe methods of follow-up  *Case-control study*—Give the eligibility criteria, and the sources and methods of case ascertainment and control selection. Give the rationale for the choice of cases and controls  *Cross-sectional study*—Give the eligibility criteria, and the sources and methods of selection of participants | 7 - 8 | *All patients were recruited between April 2018 and April 2023 from the clinic’s dedicated cardiac amyloidosis out-patient clinic. Patients were screened for eligibility and included if the predefined inclusion criteria were met: (I) a definite diagnosis of ATTR-CM, (II) consent to perform repeated assessments as described below, (III) ability to read, understand, and comply with the study requirements, (IV) evidence of at least one completed measurement of HR-QoL, and (V) a personally signed informed consent form.* |
|  |  | (*b*) *Cohort study*—For matched studies, give matching criteria and number of exposed and unexposed  *Case-control study*—For matched studies, give matching criteria and the number of controls per case |  |  |
| Variables | 7 | Clearly define all outcomes, exposures, predictors, potential confounders, and effect modifiers. Give diagnostic criteria, if applicable | 8 & 10 | *A diagnosis of ATTR-CM was established either by means of endomyocardial biopsy and subsequent congo-red staining or utilising the non-invasive diagnostic algorithm by Gillmore et al. In patients with moderate or strong cardiac 99mTechnetium-based tracer uptake in bone scintigraphy (Perugini grade ≥ 2) and exclusion of light chain amyloidosis by means of serum and urine electrophoresis and immunofixation, a diagnosis of ATTR-CM was made.”*  *“A primary endpoint of all-cause mortality was used for the present investigation. Mortality data is acquired using periodic queries on the national statistics authority (Statistik Austria), telephone interviews, online research and in- and out-of-centre medial documentation. In addition, a secondary endpoint of all-cause mortality and HF-related hospitalisations was explored using electronic health records and patient interviews. Hospitalisations were assumed to be HF-related when (1) the admission was due to dyspnoea, peripheral or central oedema (2) was resolved by HF-specific therapy and/or forced diuresis, and (3) absence of a diagnosis other than HF which the patient’s symptoms are attributed to.* |
| Data sources/ measurement | 8* | For each variable of interest, give sources of data and details of methods of assessment (measurement). Describe comparability of assessment methods if there is more than one group | 7 – 12 | Please refer to the corresponding paragraphs in the methods section |
| Bias | 9 | Describe any efforts to address potential sources of bias | 7- 12 | Throughout methods section |
| Study size | 10 | Explain how the study size was arrived at | Figure 1. | Figure 1. The patient recruitment process |

Continued on next page

| Quantitative variables | 11 | Explain how quantitative variables were handled in the analyses. If applicable, describe which groupings were chosen and why | 11 | *Categorial variables are presented as numbers and percentages while continuous variables are presented as mean and standard deviation (SD) or median and interquartile range (IQR), as appropriate. Baseline characteristics were compared using the chi-square test, Fisher’s exact test, the t-test and the Mann-Whitney-U test as applicable. For the correlation between HR-QoL scores and biomarkers of HF, the Pearson correlation coefficient (PC) was calculated.* |
| --- | --- | --- | --- | --- |
| Statistical methods | 12 | (*a*) Describe all statistical methods, including those used to control for confounding | 11 - 12 | Entire statistical analysis subsection |
|  |  | (*b*) Describe any methods used to examine subgroups and interactions | 11 - 12 | *Baseline characteristics were compared using the chi-square test, Fisher’s exact test, the t-test and the Mann-Whitney-U test as applicable.* |
|  |  | (*c*) Explain how missing data were addressed | 11 | *In case of missing data, datapoints were excluded from the respective analysis.* |
|  |  | (*d*) *Cohort study*—If applicable, explain how loss to follow-up was addressed  *Case-control study*—If applicable, explain how matching of cases and controls was addressed  *Cross-sectional study*—If applicable, describe analytical methods taking account of sampling strategy | Not applicable |  |
|  |  | (*e*) Describe any sensitivity analyses |  |  |
| Results | | | | |
| Participants | 13* | (a) Report numbers of individuals at each stage of study—eg numbers potentially eligible, examined for eligibility, confirmed eligible, included in the study, completing follow-up, and analysed | Figure 1 | Figure 1. The patient recruitment process |
|  |  | (b) Give reasons for non-participation at each stage | Figure 1 |  |
|  |  | (c) Consider use of a flow diagram | Figure 1 | Flow diagram used, please refer to figure 1. |
| Descriptive data | 14* | (a) Give characteristics of study participants (eg demographic, clinical, social) and information on exposures and potential confounders | Table 1 | Table 1. The patient cohorts’ baseline characteristics; |
|  |  | (b) Indicate number of participants with missing data for each variable of interest | Table 1 | Table 1. The patient cohorts’ baseline characteristics; Sub heading health-related quality of life |
|  |  | (c) *Cohort study*—Summarise follow-up time (eg, average and total amount) | 12 | *Patients were followed for median period of 27.6 (IQR: 9.7 – 41.8) months.* |
| Outcome data | 15* | *Cohort study*—Report numbers of outcome events or summary measures over time | 13 | *The primary endpoint of all-cause mortality occurred in 43 (25.7%) patients after a median period of 16.2 (IQR: 9.1 – 28.1) months. After 1 and 2 years, respectively, 118 (88.1%) and 90 (75.6%) of patients were alive.* |
|  |  | *Case-control study—*Report numbers in each exposure category, or summary measures of exposure |  |  |
|  |  | *Cross-sectional study—*Report numbers of outcome events or summary measures |  |  |
| Main results | 16 | (*a*) Give unadjusted estimates and, if applicable, confounder-adjusted estimates and their precision (eg, 95% confidence interval). Make clear which confounders were adjusted for and why they were included |  | Please refer to tables 3 - 6 |
|  |  | (*b*) Report category boundaries when continuous variables were categorized | Not applicable |  |
|  |  | (*c*) If relevant, consider translating estimates of relative risk into absolute risk for a meaningful time period | Not applicable |  |

Continued on next page

| Other analyses | 17 | Report other analyses done—eg analyses of subgroups and interactions, and sensitivity analyses | None performed |  |
| --- | --- | --- | --- | --- |
| Discussion | | | | |
| Key results | 18 | Summarise key results with reference to study objectives | 15 | *The results presented demonstrate that HR-QoL assessed using the EQ-5D VAS, the KCCQ, and the MLHFQ is a valid predictor of outcome in ATTR-CM. Our multivariate regression models indicate that this predictive value is independent of established biomarkers of HF in ATTR-CM.* |
| Limitations | 19 | Discuss limitations of the study, taking into account sources of potential bias or imprecision. Discuss both direction and magnitude of any potential bias | 16 | *A number of limitations of this study need to be considered. First, as with every observational study, unidentified residual confounding factors cannot be fully excluded despite scientific rigour. Due to the prospective nature of our registry, an objective primary endpoint of all-cause mortality, and event adjudication blinded to the parameters of interest, we have sought to minimise the introduction of potential bias during data analysis. However, ATTR-CM remains a disease characterised by varying presentation and severity, and the patient cohort presenting to our tertiary referral centre may be not fully representative of the entire ATTR-CM population.* |
| Interpretation | 20 | Give a cautious overall interpretation of results considering objectives, limitations, multiplicity of analyses, results from similar studies, and other relevant evidence | 15 | *The results presented demonstrate that HR-QoL assessed using the EQ-5D VAS, the KCCQ, and the MLHFQ is a valid predictor of outcome in ATTR-CM. Our multivariate regression models indicate that this predictive value is independent of established biomarkers of HF in ATTR-CM.*  *While our analysis was not designed to differentiate between distinct questionnaire scores, the EQ-5D score has demonstrated inferior predictive value with regard to all-cause mortality. The EQ-5D VAS, however, while being extremely simplistic, predicted all-cause mortality better than the complex KCCQ after adjustment for biomarkers of HF.*  *Although established in the general HF population, to the best of our knowledge, prognostic implications of HR-QoL have not yet been explored in the context of ATTR-CM specifically. Importantly, our real-world findings have implications both for disease staging and monitoring and may also aid the design of clinical trials in the future. As the tested HR-QoL questionnaires are applicable without the need for neither a physician nor extensive laboratory equipment required for the assessment of biomarkers of HF, these tools may prove valuable in the context of telehealth applications, remote disease monitoring, and early detection of disease progression and decompensation.*  *Compared to previously published ATTR-CM cohorts, our patients were slightly older, but demonstrated similar HR-QoL.5,6 In contrast to an earlier study6, we have not observed relevant differences between our ATTRwt and our ATTRv cohort with regard to HR-QoL, which may be attributed to a relatively small ATTRv cohort size.* |
| Generalisability | 21 | Discuss the generalisability (external validity) of the study results | 16 | *However, ATTR-CM remains a disease characterised by varying presentation and severity, and the patient cohort presenting to our tertiary referral centre may be not fully representative of the entire ATTR-CM population.* |
| Other information | |  | | |
| Funding | 22 | Give the source of funding and the role of the funders for the present study and, if applicable, for the original study on which the present article is based | 22 | *Funding: no external funding.* |

*Give information separately for cases and controls in case-control studies and, if applicable, for exposed and unexposed groups in cohort and cross-sectional studies.

**Note:** An Explanation and Elaboration article discusses each checklist item and gives methodological background and published examples of transparent reporting. The STROBE checklist is best used in conjunction with this article (freely available on the Web sites of PLoS Medicine at http://www.plosmedicine.org/, Annals of Internal Medicine at http://www.annals.org/, and Epidemiology at http://www.epidem.com/). Information on the STROBE Initiative is available at www.strobe-statement.org.
